# The British Journals

**Published:** 1840-10

**Authors:** 


### II. THE BRITISH JOURNALS.

(FOR THE QUARTER ENDING AUGUST 31, 1840.)

---

### EDINBURGH MEDICAL AND SURGICAL JOURNAL. JULY, 1840. No. 144.

*Observations on the Diseases of Peru.* By ARCHIBALD SMITH, M.D.

THIS is the conclusion of the paper noticed in our last Number. It contains some interesting details, particularly on the influence of climate and locality on hæmoptysis and phthisis.

---

*Cases of Great Enlargement of the Stomach; with Remarks.*
By J. H. PEEBLES, M.D., Physician to the Edinburgh Infirmary.

THIS is a monograph consisting chiefly of a collection of cases from various authors. It will be useful for reference to the practical physician.

---

*Cases of Deformity of the Feet cured by the Operation of Stromeyer.*
By ROBERT THOMSON, Esq., Surgeon, Dundee.

THE author here gives the results of fifteen cases, seven of which are detailed at length. The treatment was highly creditable to Mr. Thomson, and his paper merits the notice of surgeons.

---

*On Anomalous Affections of the Respiratory Organs.*
By JOHN GARDNER, M.D., Edinburgh.

CASES of this kind must be familiar to practitioners of experience in hysterical females of feeble frame, and who are often said to be the subjects of spinal irritation. Dr. Gardner calls attention to the employment of *blisters to the nape of the neck,* as found useful in such cases; and he suggests the same remedy, on theoretical principles but from a fair analogy, in hooping-cough and laryngismus stridulus.

*On Nervous Headach from Exhaustion, and its Treatment with Aconite.*
By T. H. Burgess, m.d.

Dr. Burgess notices two conditions of system in which nervous headach is apt to ensue; one of general anemia; the other debility from nervous exhaustion. In these, and also in other kinds of headach, he has found the extract of aconite, in doses of rather less than a grain given every two or three hours, very useful.

---

*On the Action of Hydrated Sesquioxide of Iron on Arsenic.*
By Douglas Maclagan, m.d. &c.

This is a valuable paper, and confirms the results previously arrived at of the antidotal powers of this salt of iron. Dr. Maclagan further proves that the salt acts chemically on the arsenious acid. He is of opinion that it is better fitted for removing the arsenic from its solution when precipitated by ammonia than when precipitated by potash, and that it answers better when preserved moist.

---

### Dublin Journal. July, 1840.

*On Cod-oil.* By M. Donovan, Esq.

This has been a good deal used on the continent of late years as a remedy in scrofula, (see Br. and For. Med. Review, vol. VIII. p. 554, and vol. IX. p. 553), and with supposed benefit. The present paper is valuable in showing how this medicine can be rendered palatable, which is effected by using a temperature in its expression not exceeding 192°. But Mr. Donovan's paper merits perusal.

---

*On the Utility of the Oxymuriate of Mercury in the Strumous Ophthalmia.*
By John Hamilton, Esq.

In addition to the usual local remedies, Mr. Hamilton employs with the greatest advantage this preparation of mercury internally, in doses of from one sixteenth to one eighth of a grain, in Tinct. or Decoct. Cinch., twice a day. This practice he borrowed from Sir P. Crampton, who learned it from Dr. M'Evoy. Mr. Hamilton gives several cases illustrating the great efficacy of the treatment.

---

*On the Hydrocyanoferrate of Quinina; a febrifuge of greater power than the Sulphate.*
By M. Donovan.

This remedy has been employed in Italy, and is said to answer where the ordinary salt fails. Mr. Donovan gives formulæ for the preparation and administration of the remedy.

---

### Dublin Medical Press. June—July—August, 1840.

*Direct Application of Galvanism to the Diaphragm in Suspended Animation.*
By J. Ferguson, m.d., Mullingar.

A man, who had been intoxicated when immersed, was taken out of a canal after being six or seven minutes under water, cold, livid, and lifeless. After discharging the contents of the stomach by the pump, the conductors of a galvanic battery of fifty plates were applied immediately to the diaphragm through an incision made below the seventh rib. The muscles of the chest and abdomen were instantly thrown into spasmodic action, but this subsided in a few minutes into the regular movements of respiration. Recovery ensued. *July* 1.

*On the Means of distinguishing real from apparent Enlargement of the Eyeball in cases of Hydrophthalmia and Exophthalmia.* By JAMES O'BEIRNE, M.D., Dublin.

THE following are the conclusions to which Dr. O'Beirne's examination of the subject leads: "In Hydrophthalmia the eye is completely uncovered; in Exophthalmia it is more covered than usual. In the former, the upper eyelid is parted upwards, but unchanged in all other respects; in the latter it hangs lower than usual, is more or less paralytic, and its surface is of a dusky-red colour and traversed by several enlarged veins." *Aug.* 26.

---

### LANCET. JUNE—JULY—AUGUST, 1840.

*Division of the Internal Rectus Muscle in Cases of Squinting.*
By P. BENNETT LUCAS, Esq.

OPERATED in thirty-two cases, all successful but two. *July* 4.

---

*On the Causes and Treatment of Strabismus. Summary of Seventy-six Operations by Mr. Liston.* By W. R. ANCRAM, Esq.

ALL Mr. Liston's operations were successful, in the first instance, except four; and three of these cases were cured by a second operation; the fourth would not submit to a second operation. *July* 18.

---

*Case of Hemiplegia, in which voluntary power was entirely lost, and reflex movements could be readily excited.* By W. F. BARLOW, Esq.

AN interesting case. *July* 25.

---

*Gangrene of the Mouth successfully treated by the Actual Cautery.*
By HENRY OBRE, Esq., House-surgeon to the Marylebone Infirmary.

Two cases; one cured. *Ibid.*

---

*Three Cases of Strabismus cured by Operation.* By A. T. LIGHTFOOT, Surgeon, Newcastle on Tyne. *Aug.* 1.

---

*New Mode of Operating for the Cure of Squinting.* By D. ROSS LIETCH, Esq., Surgeon, Tynemouth. *Ibid.*

---

*Cases of Strabismus cured by Operation; with Observations.*
By P. BENNETT LUCAS, Esq.

THREE more cases, thirty-eight in all, operated on by Mr. Lucas. *Aug.* 8.

---

*Abandonment of the Hook in the Operation for Squinting.*
By J. G. FRENCH, Esq.

THREE cases operated on. *Aug.* 8.

*New Hook for the Operation of Dividing the Internal Rectus Muscle of the Eye.* By J. J. Adams, Esq.

This is passed beneath the rectus at its narrowest part, and thus brings the muscle perfectly into view for incision. *Ibid.*

---

*On the Use of Sulphur in some Spasmodic Affections, particularly Angina Pectoris.* By W. Munk, m.d., Physician to the Tower Hamlets Dispensary.

Marked benefits in two cases from the use of sulphur internally, ʒss.-ʒj. once or twice daily. *July* 18.

---

*On the Use of Sulphur in Rheumatic Affections.* By Charles Clay, Esq., Surgeon, Manchester.

Mr. Clay has for several years found sulphur a valuable remedy in chronic rheumatism, used internally; and he as well as Dr. Chapman, quoted by Dr. Munk, has found its external use beneficial in the same disease and in muscular spasms. The remedy clearly deserves more notice than it commonly receives. *Aug.* 22.

---

*On the Operation for Squinting.* By Gilbert Mackmurdo, f.r.s., &c.

Mr. Mackmurdo recommends the use of the blunt hook of Mr. James Adams, which enables the operator to secure the muscle at its narrow part, and to avoid the necessity of a large incision in the conjunctiva; also the sharp acute hook of the same gentleman for fixing the eye. *Ibid.*

---

*On a New Form of Sharp Hook for fixing the Eye during the Operation for Strabismus.* By James J. Adams, Esq.

The peculiarity of this hook consists in the acuteness of turn of its sharp point, whereby pressure on the ball of the eye is avoided. *Ibid.*

---

*On Deafness and Dumbness produced by the Use of Quinine.*

In addition to several cases published before, others are related by Dr. Joseph Williams (July 25) and Mr. C. R. Bree (Aug. 22). The remedy had been given in large doses.

---

### London Medical Gazette. June—July—August, 1840.

*Observations on the Occurrence of Cerebral Disorders, in connexion with Diseased Kidneys in Children.* By Golding Bird, m.d.

This is a brief but valuable essay. In it the author carries out the views of Drs. Bright and Addison respecting the connexion between disease of the kidney and cerebral affections, and shows by the detail of two cases, the "possibility of detecting the existence of renal disease, even in very young children, by the character of the cerebral symptoms." *June* 5.

*On the Situation of the Deciduous Membrane in Cases of Extra-uterine Gestation.* By ROBERT LEE, M.D., F.R.S.

THE object of this short paper is to prove, by the relation of two cases of extra-uterine pregnancy, "that the decidua is not formed within the uterus in all cases of extra-uterine gestation." as had been generally supposed. In the two cases related, as also in one other quoted from Chaussier, the deciduous membrane was seen distinctly surrounding the ovum in the Fallopian tube. *June* 5.

---

*Case of Sero-cystic Tumours of the Breast removed by Operation; with observations on the propriety of operating in states of prostration from extreme apprehension.* By J. B. CURLING, Esq.

THIS interesting paper proves, by the relation of two cases, that even in extreme prostration of strength, capital operations may be performed with safety, and that they even act sometimes as a stimulus in rousing the energies of the system. We would, however, advise no young surgeon to incur the hazard of the dreadful alternative too probable in such circumstances. *Ibid.*

---

*On the Effects of Dividing the Inner Straight Muscle of the Eye for Strabismus.* By HERBERT MAYO, Esq.

A description of the condition of the eye six weeks after the operation. The eye operated on looks larger than the other, "from the action of the two oblique muscles not being now sufficiently antagonized." *Ibid.*

---

*On the Influence of Woollen Manufactures on Health.* By J. B. THOMSON, Surgeon, Tillicoultry.

THE author mentions, as a well-known fact, the extreme healthfulness enjoyed by the children in woollen factories, which form so striking a contrast with the state of health in cotton factories. He also adopts the popular explanation of the fact, viz., "the quantity of oils they are commonly using" in the former. The subject is curious and important, and deserves more particular investigation. *June* 12.

---

*On Dividing the Internal Rectus Muscle for the Cure of Strabismus.* By E. J. SCOTT, M.D., Portsmouth.

A successful case operated on after the manner of Mr. Lucas. *Ibid.*

---

*Cases of Strabismus.* By A. FRANZ, M.D.

THREE cases successfully operated on after Dieffenbach's method. *Ibid.*

---

*On congenital Peculiarity in the Structure of the Heart, as influencing the Diagnosis of Cardiac Affections.* By C. M. DURRANT, M.D., Ipswich.

THIS short paper will help to abate some of the difficulties of young practitioners in treating heart affections, and therefore merits perusal. It points out what every practitioner of experience knows, "that the changes arising from natural structural peculiarity often produce many of the physical signs of real disease of the organ." *June* 19.

*On the Statistics of Smallpox.* By George Gregory, m.d.

This short but interesting document gives a comparative view of the experience of Dr. Heim in Wirtemberg, and that of the author in London. Several interesting results are thus obtained: 1, that second attacks of smallpox are ten times more prevalent in Wirtemberg; 2, that the proportion of persons vaccinated is much greater in Germany than in England; 3, that the rate of mortality from primary smallpox is 36 per cent. in London, and only 20 per cent. in Germany; and 4, that the mortality of the disease after vaccination is precisely the same in both countries, viz., 7 per cent. *June* 19.

---

*On the Treatment of Croup.* By A. J. Hannay, m.d., Glasgow.

The principal and praiseworthy object of this paper is to relate the author's entire failure in the treatment of croup by sulphate of copper, a remedy recommended in an early Number of this Journal on the authority of Dr. Zimmerman. Dr. Hannay recommends, as what he has found the most successful treatment, a combination of opiates with antimonial emetics early in the disease. *July* 3.

---

*Cases illustrative of the Poisonous and Injurious Effects of the Hydriodate of Potash and Iodide of Starch.* By J. A. Lawrie, m d., Glasgow.

Seven cases are related, two of which were fatal, and the symptoms in most of the instances seem traceable to the medicine. The principal effects were affections more or less severe of the mucous membrane of the air passages, eyes, &c. with spasmodic dyspnœa, and in two cases laryngitis, intense headach, salivation, &c. The results bore no proportion to the amount of the dose, and therefore probably depended on peculiarity of constitution. At any rate they claim the earnest attention of practitioners. *Ibid.*

---

*Curious Case of Pin Swallowing.* By H. Birt, Esq., Surgeon, of Ashington, Sussex.

A girl of weak intellect, æt. twenty-three, came under the author's care in July, 1839. Before this time a surgeon had extracted twenty-seven pins from the left mamma, and in the course of four months Mr. Birt extracted 254 pins and needles (making in all 281) from almost every part of the left side of the body. The girl had been in the habit of swallowing pins and needles out of bravado or from the bribe of sweetmeats when at school almost thirteen years before. *July* 9.

---

*Two Cases of Squinting.* By A. Franz, m.d.

Two cases, requiring division of the superior oblique after that of the inner straight muscle; both cured. *July* 24.

---

*On the Use of Strychnine in Neuralgia.* By J. Pidduck, m.d., London.

Six cases of neuralgia, in its more ordinary forms and in that of sciatica, are related, in all of which a cure seemed to be speedily effected by the use of strychnia in doses of one twelfth of a grain thrice daily. *Aug.* 7.

---

*On the Operation for Strabismus.* By J. G. French, Esq.

Mr. French objects to the use of the sharp hook of Dieffenbach in fixing the eye, and uses the forceps to do this and elevate the conjunctiva for incision. Mr. French has performed the operation thrice. *Ibid.*

